# A Low-Cost Laser Interferometric Elastography System for Skin Elasticity Measurement

**DOI:** 10.3390/bioengineering13040441

**Published:** 2026-04-10

**Authors:** Asha Parmar, Shantanu Chauhan, Sora Alghziwatalkhawaldh, Kanwarpal Singh

**Affiliations:** 1 Department of Electrical and Computer Engineering, McMaster University, 1280 Main Street West, Hamilton, ON L8S 4L7, Canada; 2 Max Planck Institute for the Science of Light, 91058 Erlangen, Germany; 3 Max-Planck-Zentrum für Physik und Medizin, 91054 Erlangen, Germany

**Keywords:** laser interferometric elastography, skin elasticity, Optical Coherence Elastography

## Abstract

This paper introduces a laser interferometric elastography (LIE) system that uses a narrow linewidth laser and a single photodetector to measure mechanical displacements induced by surface acoustic waves (SAWs) generated by an electrically driven piezoelectric transducer. The method relies on phase delay analysis of the resulting interference signal to determine displacement within the medium, thereby eliminating the need for complex interferometers and broadband light sources. By substantially reducing optical hardware requirements, the system provides a compact and cost-effective platform for elasticity mapping in biological samples. Quantitative assessment of mechanical properties is achieved through controlled mechanical excitation and phase-resolved signal collection, demonstrating the practicality of simplified LIE for real-world applications.

## 1. Introduction

Measurement of the elastic properties of biological tissues is critically important in medicine, as these properties serve as valuable diagnostic biomarkers for a wide range of diseases, including cancer [1], fibrosis [2], and neurodegenerative disorders [3]. Traditionally, clinical assessment has relied on manual techniques such as palpation [4], which are inherently subjective and limited to superficial tissues. While mechanical testing methods (e.g., rheometers) can provide quantitative measurements [5], they are typically destructive and require tissue excision, making them unsuitable for in vivo applications.

To address these limitations, several non-invasive and nondestructive elastography techniques have been developed. These include optical methods such as Brillouin Microscopy [6] and Optical Coherence Elastography (OCE) [7], which enable high-resolution, microscale characterization of tissue mechanics. In addition, clinically established imaging modalities like Magnetic Resonance Elastography [8] and Ultrasound Elastography [9] provide non-invasive, quantitative assessment of tissue stiffness at organ-level depths. Together, these approaches offer a comprehensive framework for evaluating biomechanical properties across multiple spatial scales without the need for tissue removal. Among nondestructive methods, OCE, which integrates the depth-resolved imaging capability of Optical Coherence Tomography (OCT) [10,11,12,13], is emerging as a new tool for performing elastography of biological samples. Using OCE, one can achieve mechanical contrast to assess material elasticity with nanometer-scale displacement sensitivity quantitatively.

Since the introduction of OCE, the technique’s performance has been significantly influenced by the interferometric detection configuration [14]. This method has demonstrated potential for clinical applications in medical diagnostics, particularly for analyzing tissue biomechanical properties [12,15]. Stiffness or elasticity serve as key indicators of structural integrity [16,17]. OCE has been used to measure the changes in the biomechanical properties of the tissue in diseases such as systemic sclerosis and cancer, where it can be used for monitoring [11,15,18,19] the disease progression.

Interferometry-based OCE systems typically employ a broadband light source, such as a superluminescent diode (SLD), combined with an interferometric spectrometer [20,21]. This configuration enables depth-resolved optical measurements that can produce time-resolved displacement maps of biological tissues. Mechanical waves can be generated within the tissue using a piezoelectric transducer that is in contact with the sample, or using non-contact methods such as an air-puff actuator, or an ultrasound transducer [21,22,23,24]. Tissue excitation with a piezoelectric transducer results in the generation of different kinds of waves that propagate to various locations within the tissue. These include body waves, such as shear waves and pressure (longitudinal) waves, and SAWs, such as Rayleigh waves that travel along the boundary between tissue and air, and Scholte waves that propagate along the boundary between tissue and a liquid [20]. Among these, Rayleigh waves have been extensively used in OCE due to their relatively low velocities. The speed at which these induced waves travel offers an estimate of tissue stiffness, since mechanical waves propagate faster in stiffer tissues and slower in softer tissues [21]. Wave velocity is measured by analyzing the phase delay of the propagating mechanical wave or through frequency-domain dispersion analysis [25,26,27,28,29,30].

Early implementations of OCE utilized free-space interferometric configurations for tissue elasticity measurement [12,31,32,33]. However, free-space interferometric OCE systems are susceptible to surrounding disturbances, require precise optical alignment, and exhibit significant phase instability, which limits their practicality for compact, low-cost, and robust elasticity measurement platforms [12,34,35,36].

To overcome these limitations, common-path interferometric configurations were introduced, in which the reference and sample beams share nearly identical optical paths [37]. This approach substantially improves phase stability, reduces sensitivity to vibrations, and simplifies system alignment, thereby decreasing system complexity to some extent; however, it does not fully achieve low-cost implementation [34,37].

In response, the development of a laser- and detector-based common-path LIE system represents a further advancement, offering a simplified setup and reduced cost compared with free-space and interferometry-based common-path OCE systems.

In this paper, we present a method where a single photodetector can directly measure the interferometric intensity variations corresponding to phase delays induced by mechanical excitation in the sample. The displacement information is extracted through phase analysis of the interfered optical signal, eliminating the need for high-resolution imaging interferometers or complex optical setups.

This hardware-minimized approach maintains the core advantages of phase-sensitive OCE, i.e., displacement sensitivity and quantitative elasticity characterization, while offering a compact and low-cost platform. Such detector-based systems are well-suited for biomedical imaging, where portability and cost are critical.

## 2. Materials and Methods

**Figure 1:**
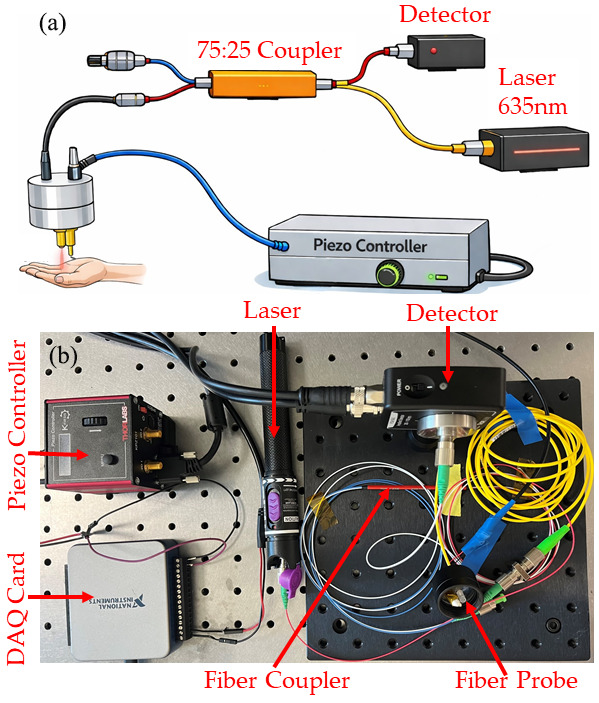
(**a**) Schematic of the common-path OCE system using a 650 nm laser, fiber coupler, ball lens probe, piezo excitation, and fixed gain detection. (**b**) Picture of the developed system.

We used a visual fault locator narrow-linewidth (1 nm) laser (Guangzhou Aicai Technology Co., Ltd, Guangzhou, China) with a central wavelength of 650 nm and output power of 29 mW. A schematic of the system is shown in Figure 1. The laser output was coupled into a 2 × 2 single-mode fiber coupler (TW670R3A2, Thorlabs, Newton, NJ, USA) with a 75:25 splitting ratio. The coupler arm with 75% laser power, delivered optical power to the common-path OCT probe (custom design). The probe consisted of a single-mode fiber terminated with a 2.5 mm diameter ball lens, which focused the beam onto the sample surface with an approximate working distance of 5 mm. Backscattered light from the sample coupled back to the same fiber and interfered with the reference reflection from the fiber–air interface. The interfered signal was detected on a fixed-gain photodetector (PDA10A2, Thorlabs, Newton, NJ, USA) through the output port of the coupler.

A piezoelectric transducer (PK2FMP1, Thorlabs, Newton, NJ, USA) was used to generate SAWs on the sample. The transducer was positioned 3 mm lateral to the optical measurement point. The distance of 3 mm between the excitation point and the measurement point was chosen to optimize the temporal delay measurement. A piezoelectric stack actuator produces a displacement of 11.2 µm when driven by a 75 V peak excitation signal at 100 Hz, with a duty cycle of 0.1% [38]. The photodetector output was digitized using a high-speed data acquisition (DAQ) card (USB 6001, National Instruments, Austin, TX 78759, USA) operating at a 20 kHz sampling rate. Continuous M-mode recordings were obtained at each measurement location, capturing temporal variations in the interferometric intensity caused by the mechanical excitation.

### 2.1. Data Processing Method

**Figure 2:**
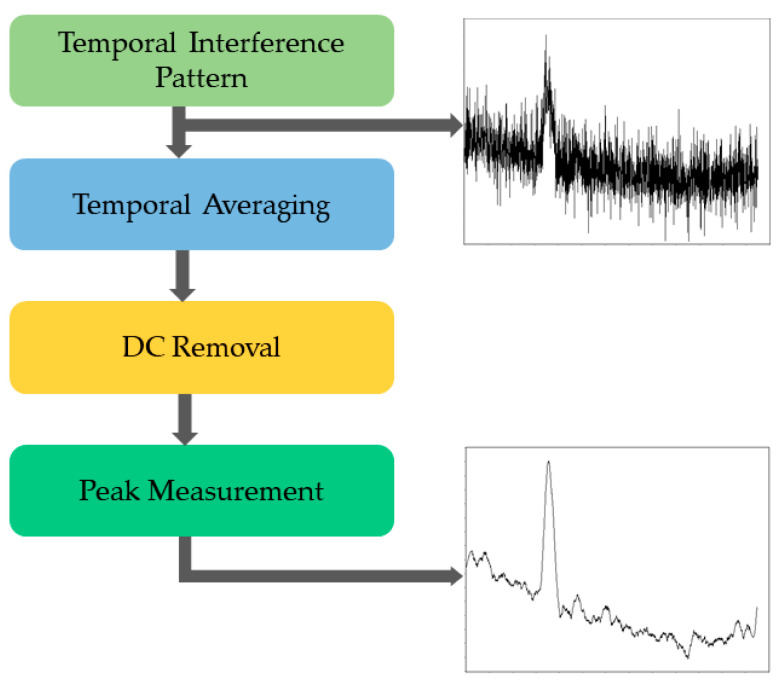
Flow chart of pulsed analysis and peak measurement.

The data processing workflow is shown in Figure 2. The temporal interference signals acquired from the detector were digitized and processed to extract displacement-related information. First, temporal averaging was applied to smooth the data. To reduce the influence of bulk tissue motion, the direct current (DC) component was removed by subtracting the mean intensity from each temporal trace. The processed signals were analyzed using a peak detection algorithm to identify the interference maxima. The positions and amplitudes of the detected peaks were extracted and used to perform a quantitative analysis of SAWs propagation. The local surface-wave velocity was then calculated using the relation:

(1) $$v=\frac{∆x}{{∆t}_{\varphi }}$$

where ∆x is the known lateral separation between measurement positions, ${∆t}_{\varphi }$ is the phase-derived temporal delay between the excitation signal and the measured signal.

Once the surface wave velocity has been obtained, the Young’s modulus of the tissue or agar phantom can be calculated by assuming that the material behaves as a homogeneous, isotropic, linear elastic medium. Under these assumptions, the Young’s modulus is related to the Rayleigh surface wave velocity and material density as follows:

(2) $$E=3.3\rho c_{r}^{2}$$

The tissue is considered to be incompressible, where ρ  is the material density taken as 1060 kg/m^3^ for soft tissue. Here, $\;c_{r}$ denotes the SAW (Rayleigh waves) velocity within the sample.

### 2.2. Sample Preparation

To validate the proposed LIE system, homogeneous agar-based tissue phantoms with a thickness of approximately 10 mm were prepared using concentrations of 1%, 2%, and 3%. These phantoms provided samples with varying stiffness levels for quantitative elasticity assessment [38]. In addition to phantom experiments, measurements were performed on human skin at representative locations, including the palm and wrist, to demonstrate in vivo applicability. All procedures involving human measurements were conducted in accordance with relevant ethical guidelines. Skin measurements were carried out as a self-evaluation by the author after providing informed consent. No additional human subjects participated in this study.

## 3. Results

**Figure 3:**
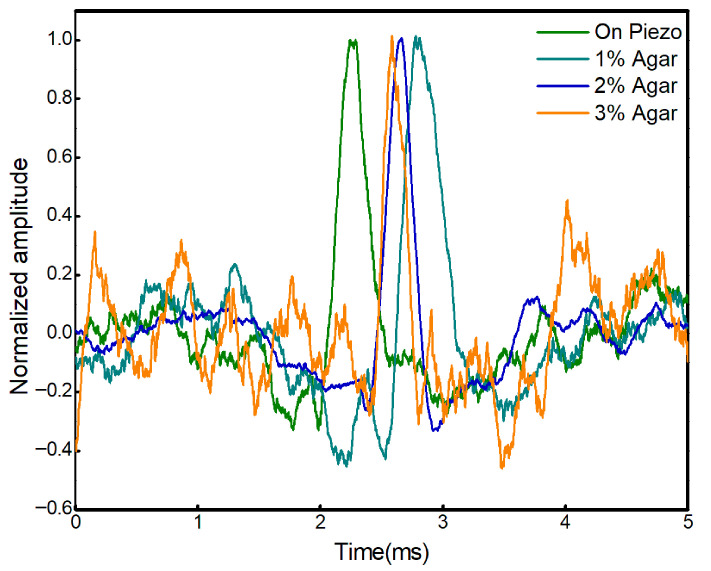
Normalized amplitude of SAW displacement measured at different agar concentration samples.

To assess the stability and performance of the proposed system, the propagation velocity of the SAWs was measured across agar phantoms with different concentrations. The temporal phase shifts extracted from the interferometric signals acquired from 1%, 2%, and 3% agar samples are presented in Figure 3. These phase shifts correspond to the propagation delay of the mechanically induced surface waves between adjacent measurement locations.

Using Equation (1), the SAWs velocities calculated for the 1%, 2%, and 3% agar phantoms were 5.5 m/s, 7.0 m/s, and 9.4 m/s, respectively. The observed increase in wave velocity with agar concentration indicates higher stiffness for phantoms with greater gel content, which is consistent with the expected mechanical behavior of agar-based tissue-mimicking materials [38].

Subsequently, the Young’s modulus values were estimated using Equation (2). The calculated Young’s modulus for the 1%, 2%, and 3% agar phantoms was 105.8 kPa, 212.8 kPa, and 309.08 kPa, respectively. These results demonstrate a monotonic increase in elasticity with agar concentration, further validating the sensitivity of the proposed LIE system.

**Figure 4:**
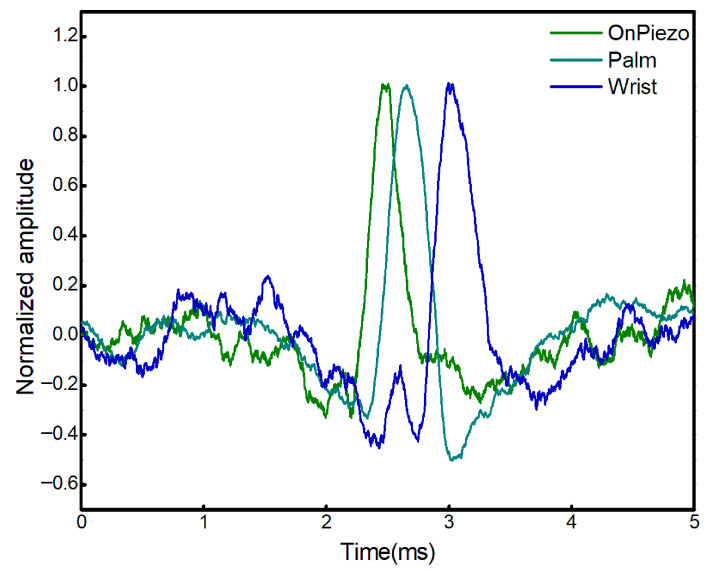
Normalized amplitude of SAW displacement measured at different positions on the hand.

Measurements were further performed at multiple laterally separated positions on human skin, as shown in Figure 4. The extracted phase-resolved displacement signals were used to determine the propagation characteristics of the SAWs. As expected, the SAWs propagated faster at the palm compared to the wrist, indicating that wave velocity increases with tissue stiffness. This behavior is consistent with the mechanical properties of skin, where the palm typically exhibits higher stiffness due to thicker epidermal and dermal layers, whereas the wrist region is comparatively more compliant. Using Equation (1), the calculated velocities at the palm and wrist are 15.6 m/s and 5.50 m/s, respectively. The calculated wave velocities for the palm and wrist exhibited trends consistent with previously reported biomechanical measurements, further validating the accuracy of the proposed method [39]. The Young’s modulus values were subsequently estimated using Equation (2). The calculated Young’s modulus at the palm and wrist was 851.2 kPa and 105.8 kPa, respectively. These results demonstrate the capability of the proposed laser- and detector-based common-path LIE system to quantitatively distinguish between tissues with different mechanical properties in vivo.

**Figure 5:**
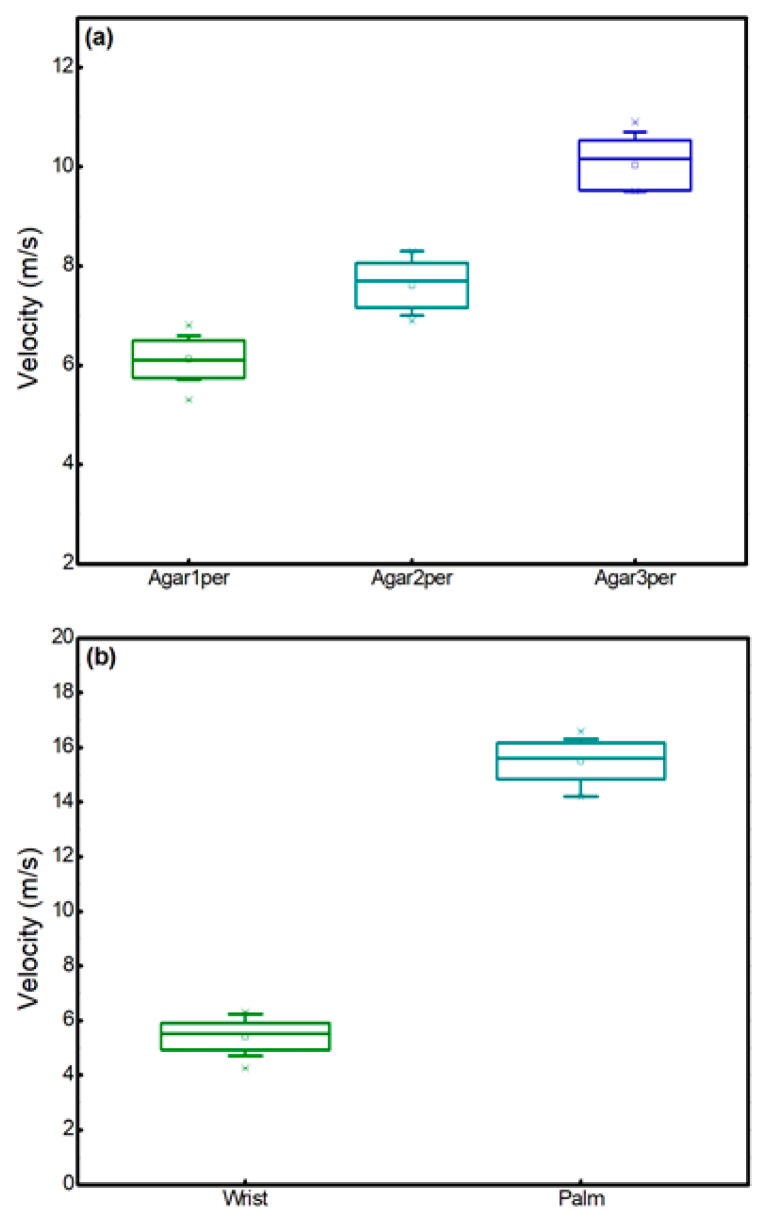
The distribution of measured SAW velocity in different samples: (**a**) represents the mean velocity at different agar concentrations (1%, 2%, and 3%); (**b**) represents the mean velocity of the skin on the palm and wrist of healthy volunteers.

The statistics of the measured velocity at different agar concentrations (1%, 2%, and 3%) for *n* = 20 at each site are shown in Figure 5a. The box plot indicates that the mean velocity for 1%, 2%, and 3% was 6.12 ± 0.38 m/s, 7.61 ± 0.44 m/s, and 10.02 ± 0.50 m/s, respectively. The increasing trend in velocity with agar concentration further confirms the expected rise in stiffness with higher gel density. The corresponding Young’s modulus values were 130.16 ± 0.31 kPa, 202 ± 0.067 kPa and 351.2 ± 0.87 kPa, respectively. The relatively small standard deviations indicate that the measurements have good repeatability and demonstrate the stability of the proposed system for quantitative elasticity estimation.

The repeatability of the measured velocity at the palm and wrist (*n* = 20 for each site), is shown in Figure 5b, demonstrating the system’s stability. The box plots indicate that the mean velocity at the wrist and the palm was 5.41 ± 0.48 m/s and 15.5 ± 0.66 m/s, respectively. The corresponding Young’s modulus values were 102.37 ± 0.80 kPa, and 840 ± 1.5 kPa.

These results demonstrate the ability of the simplified common-path LIE system to reliably quantify mechanical properties in both tissue-mimicking phantoms and the skin. Overall, the measurements represent that the proposed system provides stable and reproducible characterization of SAWs propagation and tissue elasticity while significantly reducing system complexity compared to traditional camera-based OCT configurations.

A cost analysis of the proposed system was performed and is summarized in Table 1.

## 4. Discussion

In this work, we developed a simplified and cost-effective common-path LIE system capable of detecting SAWs using only a narrow-linewidth laser, a fiber-based probe, and a single photodetector. The key advantage of the system is its significantly reduced hardware complexity and cost compared with conventional camera-based OCT elastography systems.

As summarized in Table 1, this simplified architecture reduces the overall system cost by nearly an order of magnitude while maintaining a reliable measurement capability for SAWs-based elasticity characterization.

The system successfully measured phase delays and surface-wave velocities across multiple positions on the sample surface, demonstrating a performance comparable to conventional interferometric OCE systems while significantly reducing optical complexity, alignment requirements, and cost. The use of DC removal and temporal averaging in processing the interferometric signals ensured that bulk tissue motion and background intensity were effectively suppressed. This, combined with peak detection for identifying interference maxima, allowed for robust and repeatable extraction of phase-resolved displacements.

A potential limitation of the present system is that surface curvature was not explicitly incorporated into the wave propagation analysis. In this study, measurements were performed along the longitudinal direction of the wrist; therefore, the propagation distance was estimated assuming a locally flat surface along this direction. However, the wrist exhibits spatial curvature, and deviations from planarity may alter the true propagation path length. As a result, differences in measured arrival times may partially reflect geometric effects associated with local curvature rather than purely variations in tissue elasticity.

Additionally, variations in the axial distance from the skin surface to underlying rigid structures (e.g., bones) were not explicitly accounted for. The analysis assumes a semi-infinite homogeneous soft-tissue medium, whereas the soft-tissue thickness over the wrist can vary along the measurement direction. Changes in the depth to rigid boundaries may influence wave propagation through boundary interactions, reflections, or altered dispersion characteristics. These effects may introduce bias in the estimated wave speed, particularly in regions where the soft-tissue layer is thinner.

Although the measurements were acquired along the length of the wrist to reduce curvature-related effects compared to transverse directions, residual curvature and depth variations may still influence the results and should therefore be considered as a potential limitation of the current approach.

Overall, this work establishes a strong foundation for the development of compact, portable, and clinically deployable OCE devices. Future efforts will focus on enabling two-dimensional elasticity mapping and validating the system across diverse biological tissues.

The reduced hardware requirements also improve the system’s portability and ease of implementation, making the technique attractive for cost-sensitive biomedical and clinical applications where compact and affordable instrumentation is desirable.

## 5. Patents

The authors have filed for a provisional patent for the technique described in the manuscript (United States of America Patent Application No: 63/992493).

## Figures and Tables

**Table 1:** System estimated cost.

Items	Vender	Cost ($)
1. 650 nm wavelength Laser	Guangzhou Aicai Technology Co., Ltd., Guangzhou, China	30.00
2. 2 × 2 single-mode fiber coupler	Thorlabs, Newton, NJ, USA	453.78
3. Single-mode fiber	FS, New Castle, DE, USA	10.00
4. Ball lens	Uxcell, Hong Kong, China	3.00
5. Single-mode photodiode	Thorlabs, Newton, NJ, USA	385.14
6. Piezo transducer	Thorlabs, Newton, NJ, USA	114.39
7. Piezo controller	Thorlabs, Newton, NJ, USA	800.00
8. DAQ Card	National Instruments, Austin, TX, USA	450.00
	Total (Canadian dollar)	2246.31

## Data Availability

The data that support the findings of this study are available from the corresponding author upon reasonable request.
